# Pemafibrate is associated with greater reductions in MDA-LDL and small dense LDL fractions with exploratory PBMC gene-expression changes: a prespecified subanalysis of the PARADISE in Kagoshima study

**DOI:** 10.3389/fcdhc.2026.1850470

**Published:** 2026-07-01

**Authors:** Toru Kubo, Takahisa Deguchi, Kaori Matsuzaki, Atsushi Shinnakasu, Shigeru Kawade, Aiko Arimura, Hiroshi Hashiguchi, Kazuma Ogiso, Tetsuro Kamada, Mihoko Kurano, Katsutaro Morino, Yoshihiko Nishio

**Affiliations:** 1 Department of Diabetes and Endocrine Medicine, Kagoshima University Graduate School of Medical and Dental Sciences, Kagoshima, Japan; 2 Department of Medicine, Izuro Imamura Hospital, Kagoshima, Japan; 3 Hayato Medical Clinic, Kirishima, Japan; 4 Gyokusuikai Hospital, Kagoshima, Japan; 5 National Hospital Organization Kagoshima Medical Center, Kagoshima, Japan

**Keywords:** eicosapentaenoic acid, gene expression, MDA-LDL, pemafibrate, peripheral blood mononuclear cells, small dense LDL

## Abstract

**Background:**

Pemafibrate, a selective peroxisome proliferator-activated receptor alpha (PPARα) modulator, lowers triglycerides and improves lipid parameters. However, its effects on oxidized LDL-related markers, including malondialdehyde-modified low-density lipoprotein (MDA-LDL), and associated *in vivo* gene-expression changes in humans remain limited. In addition, whether pemafibrate-associated changes in oxidized LDL-related biomarkers have clinical implications for atherosclerotic progression or cardiovascular risk remains uncertain.

**Methods:**

In this prespecified subanalysis of the randomized, open-label, parallel-group PARADISE in Kagoshima study, 51 participants assigned to pemafibrate and 46 assigned to eicosapentaenoic acid (EPA) were included in the clinical analysis. All participants had type 2 diabetes and hypertriglyceridemia and received treatment for 16 weeks. We evaluated changes in MDA-LDL, lipoprotein fractions including small dense LDL-related fractions, and exploratory peripheral blood mononuclear cell (PBMC) gene expression. PBMC gene expression was assessed in 11 participants in the Pemafibrate group and 7 in the EPA group, and was considered exploratory and hypothesis-generating.

**Results:**

Compared with EPA, pemafibrate produced a significantly greater reduction in triglycerides (least-squares mean difference, −79.2 mg/dL; 95% confidence interval [CI], −117 to −41.5; P < 0.001), and a greater increase in HDL-C (4.64 mg/dL; 95% CI, 2.43 to 6.84; P < 0.001). The mean change in MDA-LDL from baseline to week 16 was −30.3 ± 32.4 U/L in the Pemafibrate group and −9.2 ± 44.3 U/L in the EPA group, yielding a least-squares mean difference of −21.2 U/L (95% CI, −36.9 to −5.4; P = 0.009). In the exploratory PBMC analysis, ABCA1 and LCAT remained significant after false discovery rate adjustment, whereas other genes showed only nominal differences.

**Conclusion:**

Pemafibrate was associated with greater 16-week reductions in MDA-LDL and small dense LDL fractions than EPA in patients with type 2 diabetes and hypertriglyceridemia. These findings suggest modification of selected lipid-quality biomarkers. Their clinical relevance remains to be established.

## 1 Introduction

Patients with diabetes are prone to hypertriglyceridemia because insulin resistance promotes hepatic overproduction of very-low-density lipoprotein (VLDL), a triglyceride-rich lipoprotein (1). Hypertriglyceridemia is increasingly recognized as a component of residual atherosclerotic cardiovascular risk. This concept is supported by a systematic review and meta-analysis showing that elevated triglyceride levels are associated with increased cardiovascular disease risk in patients with type 2 diabetes, as well as by cohort data demonstrating that triglyceride levels predict residual cardiovascular risk even in statin-treated patients with newly diagnosed type 2 diabetes (2, 3). In addition, triglyceride-rich lipoprotein metabolism promotes an atherogenic lipoprotein profile characterized by increased remnant lipoproteins and small dense low-density lipoprotein (LDL) particles (4–6). HDL-cholesterol (HDL-C) levels are also often reduced in diabetes, and HDL particles tend to become smaller, further increasing cardiovascular risk (7).

Pemafibrate is a selective peroxisome proliferator-activated receptor alpha (PPARα) modulator. Compared with conventional fibrates such as bezafibrate and fenofibrate, it has greater potency and selectivity for PPARα activation, and clinical studies have shown that it effectively lowers triglyceride levels and increases HDL-C levels at low doses (8). These lipid-modifying effects suggest that pemafibrate may improve not only quantitative lipid abnormalities but also qualitative changes in atherogenic lipoproteins and oxidized lipoprotein markers.

Malondialdehyde-modified LDL (MDA-LDL) is a representative form of oxidized LDL generated by malondialdehyde modification of apolipoprotein B. It reflects enhanced oxidative stress and promotes macrophage foam cell formation through scavenger receptors, thereby contributing to atherogenesis independently of LDL-cholesterol levels (9, 10). MDA-LDL has also been associated with coronary artery disease and atherosclerotic burden in observational studies (11, 12). Previous studies have shown that bezafibrate and fenofibrate reduce not only triglycerides but also MDA-LDL levels (13, 14). In contrast, the MDA-LDL-lowering effect of pemafibrate has not been well documented.

This question is clinically relevant because the cardiovascular implications of triglyceride-lowering therapies appear to differ according to the agent used. In large-scale clinical trials, EPA-based therapy reduced major cardiovascular events, whereas pemafibrate, despite improving triglycerides and other lipid parameters, did not reduce cardiovascular events in statin-treated patients with type 2 diabetes and atherogenic dyslipidemia (15–17). These findings indicate that improvements in triglycerides and related lipid biomarkers should not be equated with proven cardiovascular outcome benefit. Nevertheless, they raise the possibility that pemafibrate and EPA may differ in their effects on lipid-quality biomarkers and peripheral cellular responses. In particular, their effects on small dense LDL, oxidized LDL such as MDA-LDL, and treatment-related cellular responses may differ. Therefore, comparing pemafibrate and EPA may help characterize differences in lipid-quality biomarkers, but cannot establish clinical superiority of one treatment over the other.

In an experimental study using primary human hepatocytes, pemafibrate induced the expression of multiple genes involved in lipid metabolism, including ABCA1, FGF21, and VLDLR (18). However, evidence regarding *in vivo* gene-expression changes in humans remains scarce, and no study has directly examined whether differences in peripheral blood mononuclear cell (PBMC) gene expression between pemafibrate and EPA are associated with changes in MDA-LDL. PBMCs are readily accessible and may provide exploratory information on peripheral immune-metabolic and cholesterol transport-related responses. Therefore, using EPA as an active comparator, we examined whether pemafibrate was associated with differential changes in small dense LDL fractions, MDA-LDL, and exploratory PBMC gene expression in patients with type 2 diabetes and hypertriglyceridemia. The aim of this prespecified subanalysis was to characterize lipid-quality biomarker changes and generate hypotheses regarding PBMC gene-expression changes related to cholesterol handling, with particular attention to exploratory signals related to monocyte/macrophage-lineage cholesterol handling and their association with MDA-LDL reduction.

## 2 Methods

We evaluated the effect of pemafibrate on FMD in patients with type 2 diabetes and hypertriglyceridemia in a randomized parallel-group comparative trial (PARADISE in Kagoshima study: jRCTs#071190049) and reported non-inferiority. In that study, 100 patients attending Kagoshima University Hospital and Izuro Imamura Hospital between June 2020 and October 2021 were randomized to either the Pemafibrate group or the comparator eicosapentaenoic acid (EPA) group and followed for 16 weeks. This study presents a subanalysis examining changes in MDA-LDL and PBMC gene expression.

### 2.1 Study design and ethical approval

The present study was a prespecified subanalysis of the PARADISE in Kagoshima study, a multicenter, randomized, open-label, parallel-group comparative trial that evaluated the effect of pemafibrate on flow-mediated dilation (FMD) in patients with type 2 diabetes and hypertriglyceridemia and demonstrated non-inferiority versus eicosapentaenoic acid (EPA). The parent trial was registered in the Japan Registry of Clinical Trials (jRCTs071190049) on March 6, 2020. A total of 100 patients attending Kagoshima University Hospital and Izuro Imamura Hospital between June 2020 and October 2021 were randomized to either the Pemafibrate group or the EPA group and followed for 16 weeks. The present analysis examined changes in MDA-LDL and PBMC gene expression. The study protocol was approved by the Institutional Review Board of Kagoshima University Hospital (first approval: June 28, 2019; approval No. 19-K14; second approval for protocol extension: March 3, 2025; approval No. 240187epi). Written informed consent was obtained from all participants before enrollment. The study was conducted in accordance with the Declaration of Helsinki and the Clinical Trials Act in Japan.

### 2.2 Participants

Eligible participants were outpatients with type 2 diabetes and hypertriglyceridemia, defined as casual triglyceride (TG) levels of 175–500 mg/dL or fasting TG levels of 150–500 mg/dL. The exclusion criteria were as follows: type 1 diabetes; poor glycemic control (HbA1c ≥9.0% within the previous 6 months); renal impairment (serum creatinine ≥1.5 mg/dL); hypersensitivity or contraindications to either study drug; use of fibrate-class drugs within 4 weeks before consent; use of omega-3 fatty acid preparations or equivalent supplements within 6 months before consent; use of corticosteroids such as prednisolone within 6 months before consent; changes in the dosage of HMG-CoA reductase inhibitors or angiotensin II receptor blockers within 4 weeks before consent; active cancer or ongoing anticancer chemotherapy; bleeding tendency or active bleeding; familial hypercholesterolemia; pregnancy, possible pregnancy, or breastfeeding; current participation in another interventional trial; and any condition judged by the treating physician to make the patient unsuitable for the study.

### 2.3 Randomization and interventions

After confirmation of eligibility and acquisition of written informed consent, participants were randomized electronically by an external trial office using a centralized web-based system. Assignment was performed centrally through a web-based system inaccessible to investigators before enrollment. Randomization was performed in a 1:1 ratio using the minimization method, with HbA1c, body mass index (BMI), and TG level at the time of consent as allocation factors. Participants assigned to the Pemafibrate group received pemafibrate 0.1 mg orally twice daily (morning and evening). Participants assigned to the EPA group received ethyl eicosapentaenoic acid 1800 mg/day, administered as either 900 mg twice daily or 600 mg three times daily immediately after meals. Follow-up lasted 16 weeks (± 2 weeks), and clinical and laboratory assessments were performed at baseline and at week 16. In the parent trial, 52 participants were assigned to the Pemafibrate group and 48 to the EPA group. PBMC samples were collected from most participants; however, the number of samples available for gene expression analysis was limited by RNA quantity and quality. Ultimately, 11 participants in the Pemafibrate group and 7 in the EPA group were included in the PBMC gene expression analysis.

### 2.4 Sample size

The sample size of the parent trial was determined based on the primary endpoint, FMD. Based on previous studies, we assumed that the change in FMD would be 2.0% in the Pemafibrate group and 1.0% in the EPA group, with a standard deviation of 1.6% in both groups. With a two-sided alpha level of 0.05, 80% power, and an anticipated dropout rate of 10%, 47 participants per group were required. Therefore, the target sample size was set at 100 participants in total. No separate sample size calculation was performed for the present subanalysis, which was considered exploratory. For the PBMC gene-expression substudy, a *post-hoc* power/sensitivity assessment was performed. With 11 participants in the Pemafibrate group and 7 in the EPA group, a two-sided two-sample comparison with α = 0.05 and 80% power would require a standardized effect size of approximately 1.44.

### 2.5 Outcome measures for the present subanalysis

The outcomes of the present subanalysis were changes from baseline to week 16 in MDA-LDL, remnant-like particle cholesterol (RLP-C), urinary 8-hydroxy-2′-deoxyguanosine (8-OHdG), high-sensitivity C-reactive protein (hs-CRP), conventional lipid parameters, lipoprotein fractions including small dense LDL-related fractions, and PBMC mRNA expression levels. The association between these variables and changes in MDA-LDL was further explored using SEM. All PBMC gene-expression and SEM analyses were considered exploratory and hypothesis-generating.

### 2.6 Laboratory measurements

All blood and urine samples were collected after an overnight fast of at least 10 h. Serum levels of RLP-C, MDA-LDL, hs-CRP, TG, total cholesterol (TC), HDL-C, and LDL-C, as well as urinary 8-OHdG normalized to creatinine, were measured centrally by SRL, Inc. (Tokyo, Japan), in order to minimize inter-institutional variability. Most blood and urine samples were analyzed on the day of collection. Lipoprotein fractions were analyzed by high-performance liquid chromatography using the LipoSEARCH system at Skylight Biotech Inc. (Tokyo, Japan).

### 2.7 PBMC isolation and RNA extraction

Peripheral blood samples (10 mL) were collected into EDTA-coated tubes. Samples were diluted 1:1 with phosphate-buffered saline (PBS), layered over 10 mL of Lymphoprep (Axis-Shield, Oslo, Norway), and centrifuged at 800 × g for 20 min. Mononuclear cells were collected from the interface layer. The PBMC pellet was homogenized in 0.75 mL TRIzol reagent (Life Technologies) and incubated for 5 min at room temperature. After addition of 0.2 mL chloroform, the homogenate was centrifuged at 12,000 × g for 15 min at 4 °C using a microcentrifuge (Eppendorf 5417R). The aqueous phase was transferred to a new tube, and RNA was precipitated with 0.5 mL isopropanol, followed by centrifugation at 12,000 × g for 10 min at 4 °C. The RNA pellet was washed with 1 mL of 75% ethanol, centrifuged at 7,500 × g for 5 min at 4 °C, air-dried, and dissolved in 100 μL RNase-free water. PBMC samples were stored at -80 °C until analysis. RNA concentration and purity were assessed spectrophotometrically using a NanoDrop 1000 (Thermo Fisher Scientific, USA). Samples with an A260/A280 ratio of 1.70-2.00 were considered acceptable for analysis.

### 2.8 Quantitative real-time RT-PCR

RNA extraction and quantitative real-time RT-PCR were performed at Kagoshima University. Single-stranded cDNA was synthesized from 1 μg total RNA using the High Capacity cDNA Reverse Transcription Kit (Applied Biosystems). Reverse transcription was performed at 37 °C for 2 h, followed by denaturation at 85 °C for 5 min using Power BLOCK (ATTO, Japan). Quantitative real-time PCR was performed on a StepOnePlus Real-Time PCR System (Applied Biosystems) according to the manufacturer’s instructions. Each reaction contained 100 ng of cDNA in a final volume of 20 μL, including TaqMan qPCR master mix and TaqMan Gene Expression Assays (Applied Biosystems; **Supplementary Table 1**). Reactions were run in duplicate in 96-well plates under the following conditions: 95 °C for 20 s, followed by 40 cycles of 95 °C for 1 s and 60 °C for 20 s. Based on previous studies, we selected genes related to inflammation, lipid metabolism, and atherosclerosis: NFKB1, TNFA, IL1B, CCL2, IL6, HMOX1, LPL, ABCA1, ABCG1, SCARB1, LCAT, CETP, PPARA, CD36, EDN1, ICAM1, and PAFR. Primer/probe information is provided in **Supplementary Table 1**. Gene expression levels were normalized to HPRT1 as the endogenous control. Relative mRNA expression levels were analyzed using the ΔCt and ΔΔCt methods.

### 2.9 Statistical analysis

Clinical data were analyzed with support from Soiken, Inc. (Osaka, Japan). Continuous variables are expressed as mean ± standard deviation, and categorical variables as number (percentage). Baseline characteristics were compared between groups using the unpaired t test for continuous variables and Fisher’s exact test for categorical variables. Within-group changes from baseline to week 16 were evaluated using the paired t test. Between-group differences in changes from baseline were assessed using analysis of covariance (ANCOVA), with the baseline value of each variable included as a covariate. Least-squares mean differences with 95% confidence intervals (CIs) were calculated. ANCOVA assumptions including homogeneity of regression slopes and linearity of responses, were assessed (**Supplementary Table 2**), and complementary analyses using change scores and unpaired t-tests were also performed (**Supplementary Table 3**). A two-sided P value <0.05 was considered statistically significant. Because the present subanalysis, including gene expression analysis, was exploratory, raw P-values were calculated for between-group comparisons, and Benjamini–Hochberg false discovery rate-adjusted q-values were additionally calculated for the 17 PBMC gene-expression markers. Both raw P-values and FDR-adjusted q-values are reported. The PBMC gene-expression findings were interpreted as exploratory and hypothesis-generating. Analyses were performed using complete-case data. Baseline characteristics were compared between participants included in and excluded from the PBMC gene-expression substudy to assess potential selection bias related to RNA availability and quality. Sensitivity analyses stratified by baseline statin use were performed for MDA-LDL outcome where feasible. These analyses were descriptive and exploratory, and formal treatment-by-statin interaction tests were not performed. Correlation coefficients and P-values were also calculated for the associations between changes in MDA-LDL and changes in each PBMC gene-expression marker. To explore structural relationships among lipid metabolism markers, PBMC gene expression, and oxidative stress, we performed structural equation modeling (SEM) separately in the pemafibrate and EPA groups. The hypothesized model included paths from PPARA to LPL, from LPL to TG, from TG to small dense LDL, and from small dense LDL and selected cholesterol transport-related genes (ABCA1, ABCG1, LCAT, and SCARB1) to MDA-LDL. A covariance between ABCA1 and ABCG1 was added based on modification indices to improve model fit. Given the limited sample size and the exploratory nature of this subanalysis, SEM was used only as a visual and hypothesis-generating approach and was not intended to establish a confirmatory mechanistic model. Model fit was assessed using the comparative fit index (CFI), Tucker-Lewis index (TLI), root mean square error of approximation (RMSEA), and standardized root mean square residual (SRMR). A part of ANCOVA and all SEM analyses were performed using Exploratory v15.3 (Exploratory, Inc., USA, https://exploratory.io 32), R version 4.4.0 with the lavaan package, and path diagrams were generated using the semPlot package. Standardized coefficients were used for path visualization to facilitate direct comparison between the two groups.

## 3 Results

### 3.1 Baseline clinical characteristics of the study participants

**Figure 1:**
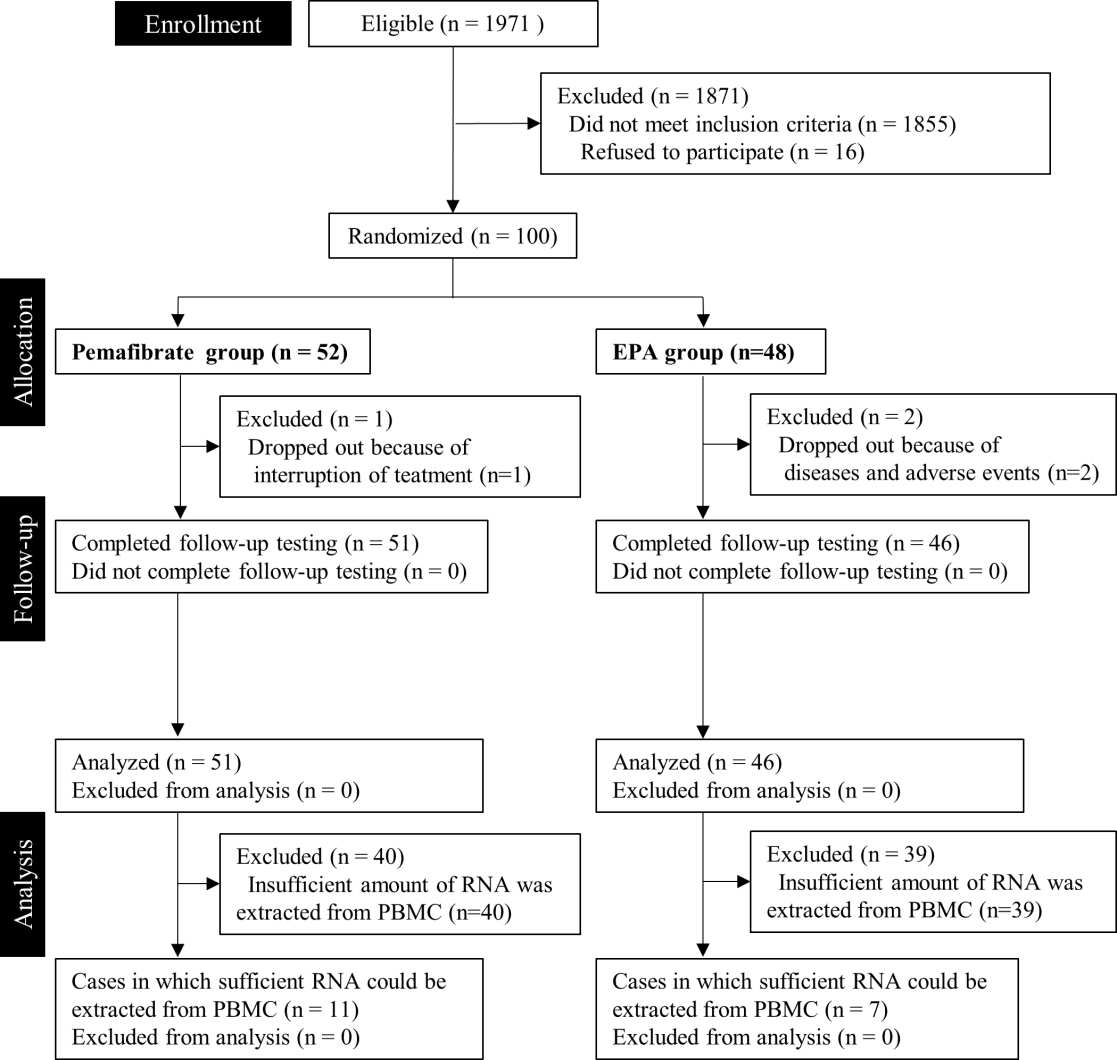
Flow diagram of participant selection and study completion. Outpatients with type 2 diabetes and hypertriglyceridemia, defined as casual triglyceride levels of 175-500 mg/dL or fasting triglyceride levels of 150-500 mg/dl, were screened for eligibility. After application of the exclusion criteria and acquisition of written informed consent, eligible participants were randomized to the pemafibrate group or the eicosapentaenoic acid (EPA) group and followed for 16 weeks. The figure also shows the subset of participants included in the PBMC gene expression analysis. PBMC, peripheral blood mononuclear cells.

A total of 1971 potentially eligible patients with type 2 diabetes mellitus and hypertriglyceridemia were identified at Kagoshima University Hospital and Izuro Imamura Hospital between May 2020 and October 2021, and 100 were enrolled in the study. **Figure 1** shows the participant flow. The participants were randomly assigned to the Pemafibrate group (n = 52) or the EPA group (n = 48). As shown in **Figure 1**, one patient in the Pemafibrate group discontinued follow-up, and two patients in the EPA group discontinued the study because of another illness and an adverse event, respectively. Consequently, 97 patients completed the study. **Table 1** summarizes the baseline demographic and clinical characteristics of the study participants. Baseline characteristics were comparable between the two groups.

**Table 1:** Demographic and baseline characteristics of the participants.

Characteristic	Total (n=97)	Pemafibrate group (n=51)	EPA group (n=46)	*P*-value
Age (years)	57.1 ± 10.7	57.6 ± 9.2	56.5 ± 12.2	0.61
Gender (male/female)	59/38	30/21	29/17	0.68
Height (cm)	163.6 ± 8.6	162.3 ± 8.6	165.0 ± 8.5	0.11
Body weight (kg)	76.6 ± 18.5	75.4 ± 19.8	77.9 ± 17.0	0.50
Body Mass Index (kg/m^2^)	28.5 ± 5.8	28.5 ± 6.5	28.5 ± 5.1	1.00
Systolic BP (mmHg)	128.7 ± 13.9	127.2 ± 12.6	130.5 ± 15.3	0.24
Diastolic BP (mmHg)	80.3 ± 11	80.0 ± 11.4	80.7 ± 10.6	0.75
HbA1c (mmol/mol, %)	52.4 ± 7.7, 6.9 ± 0.7	53.2 ± 6.8, 7.0 ± 0.6	51.4 ± 8.6, 6.9 ± 0.8	0.25
eGFR (mL/min/1.73 m^2^)	76.8 ± 19.6	76.8 ± 19.4	76.9 ± 20.1	0.98
Duration of type 2 diabetes mellitus (years)	9 ± 8.6	9.0 ± 7.7	9.0 ± 9.6	0.99
Diabetes-related disease [n(%)]				
Retinopathy	25 (32.5)	12 (30.8)	13 (34.2)	0.81
Nephropathy	27 (28.7)	14 (29.2)	13 (28.3)	1.00
Neuropathy	20 (23.8)	9 (20.9)	11 (26.8)	0.61
Medications [n(%)]				
Metformin	86 (88.7)	43 (84.3)	43 (93.5)	0.21
DPP-4 inhibitor	56 (57.7)	31 (60.8)	25 (54.3)	0.54
Sulfonylurea	7 (7.2)	4 (7.8)	3 (6.5)	1.00
Thiazolidinedione	9 (9.3)	7 (13.7)	2 (4.3)	0.16
α-GI	13 (13.4)	7 (13.7)	6 (13.0)	1.00
Glinide	9 (9.3)	5 (9.8)	4 (8.7)	1.00
SGLT2 inhibitor	46 (47.4)	25 (49.0)	21 (45.7)	0.84
GLP1 recepter agonists	16 (16.5)	9 (17.6)	7 (15.2)	0.79
Anti-hypertensive medication	58 (59.8)	32 (62.7)	26 (56.5)	0.54
Anti-hyperlipidemic medication	45 (46.4)	25 (49.0)	20 (43.5)	0.68
Hypertension [n(%)]	57 (58.8)	31 (60.8)	26 (56.5)	0.69
Smoking [n (%)]				0.77
Never	51 (52.6)	28 (54.9)	23 (50.0)	
Former	20 (20.6)	11 (21.6)	9 (19.6)	
Current	26 (26.8)	12 (23.5)	14 (30.4)	

Values are mean ± SD for continuous variables. The *P* values indicate the difference between the Pemafibrate group and the EPA group using the Fisher’s exact test.

### 3.2 Lipid and oxidative stress marker analysis

**Figure 2:**
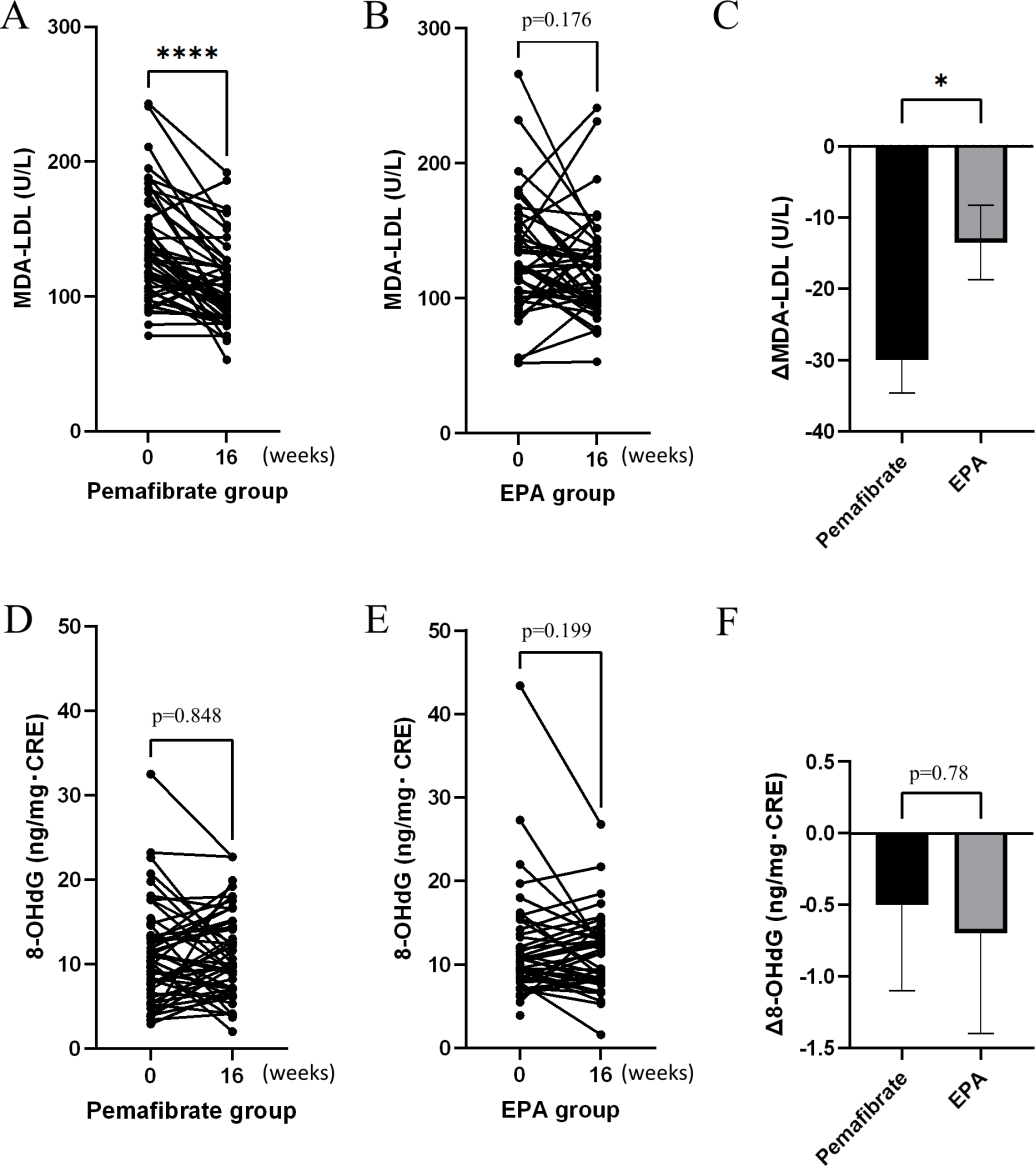
Changes of MDA-LDL. and 8-OHdG in the study group. **(A, B, D, E)** Data are means. The p-value is obtained by paired T-test between before (0 week) and after (16 week) intervention. ****P<0.0001. **(C, F)** Data are means ± SD. The data is obtained by using an analysis of covariance with group as a fixed effect, assignment adjustment factors, and corresponding baseline values as covariates. *P<0.05.

As shown in **Table 2**, pemafibrate produced a significantly greater reduction in TG than EPA [change, -92.0 ± 98.1 *vs*. -17.3 ± 82.3 mg/dL; least-squares mean difference, -79.2 mg/dL; 95% confidence interval (CI), -117 to -41.5; P < 0.001]. HDL-C also increased to a significantly greater extent in the Pemafibrate group than in the EPA group (change, 4.5 ± 7.3 *vs*. -0.4 ± 4.8 mg/dL; least-squares mean difference, 4.64 mg/dL; 95% CI, 2.43 to 6.84; P < 0.001). Among lipoprotein fractions, pemafibrate was associated with significantly greater reductions in VLDL-C (least-squares mean difference, -12.0 mg/dL; 95% CI, -16.4 to -7.51; P < 0.001), VLDL-TG (-70.4 mg/dL; 95% CI, -99.3 to -41.5; P < 0.001), HDL-TG (-3.11 mg/dL; 95% CI, -4.83 to -1.38; P < 0.001), and RLP-C (-3.50 mg/dL; 95% CI, -5.51 to -1.49; P < 0.001) than EPA. LDL-C and LDL-TG did not differ significantly between groups. The reduction in MDA-LDL was also greater in the Pemafibrate group than in the EPA group. The mean change in MDA-LDL from baseline to week 16 was -30.3 ± 32.4 U/L in the Pemafibrate group and -9.2 ± 44.3 U/L in the EPA group (**Figures 2A, B**). The least-squares mean difference in change in MDA-LDL between groups was -21.2 U/L (95% CI, -36.9 to -5.4; P = 0.009) (**Figure 2C**). Because baseline MDA-LDL was numerically higher in the Pemafibrate group, this between-group comparison was adjusted for baseline MDA-LDL using ANCOVA. Urinary 8-OHdG showed no significant change in either group (**Figures 2D, E**), and the least-squares mean difference between groups was not significant (**Figure 2F**). Similarly, hs-CRP did not differ significantly between groups (least-squares mean difference, 0.019 mg/dL; 95% CI, -0.052 to 0.090; P = 0.59; **Table 2**). Sensitivity analyses stratified by baseline statin use were performed for changes in MDA-LDL. The reduction in MDA-LDL with pemafibrate appeared numerically greater among statin users than among non-users; however, these subgroup findings were descriptive and limited by small sample size. Formal treatment-by-statin interaction tests were not performed. These findings are summarized in **Supplementary Figure 1**. Fasting plasma glucose, HbA1c, body weight, serum creatinine, and creatine phosphokinase also showed no significant between-group differences (**Table 2**). In contrast, liver-related parameters improved in the Pemafibrate group compared with the EPA group, with significant least-squares mean differences for AST (-6.15 U/L; 95% CI, -11.9 to -0.372; P = 0.04), ALT (-13.7 U/L; 95% CI, -26.4 to -1.08; P = 0.03), and γ-GT (-17.9 U/L; 95% CI, -27.2 to -8.67; P < 0.001) (**Table 2**). Complementary analyses using change scores showed results generally consistent with the primary ANCOVA findings (**Supplementary Table 3**).

**Table 2:** Changes in parameters between baseline and week 16 in each treatment group.

Characteristic	Pemafibrate group (n=51)			EPA group (n=46)			Difference in changes (95% CI)	*P* value
	Baseline	Week 16	Change	Baseline	Week 16	Change		
Body Weight (kg)	75.4 ± 19.8	75.2 ± 19.5	-0.2 ± 1.8	77.9 ± 17.0	78.7 ± 17.4	0.3 ± 1.6	-0.472 (-1.174, 0.230)	0.18
AST (units/L)	28.1 ± 13.6	26.5 ± 13.5	-1.2 ± 12.1	26.0 ± 9.2	31.2 ± 16.7	5 ± 16	-5.60 (-11.1, -0.06)	0.048
ALT (units/L)	35.2 ± 21.6	29.4 ± 22.6	-5.8 ± 16.4*	32.5 ± 17.2	41.2 ± 41.5	7.9 ± 42	-12.9 (-25.4, -0.53)	0.041
γGT (units/L)	44.0 ± 32.8	29.2 ± 25.5	-14.7 ± 15.2***	53.2 ± 37.8	57.4 ± 43.2	3.2 ± 29	-20.2 (-29.0, -11.4)	<0.001
sCr (mg/dL)	0.77 ± 0.20	0.79 ± 0.21	0.02 ± 0.07	0.78 ± 0.20	0.77 ± 0.20	0.02 ± 0.06	0.026 (-0.003, 0.054)	0.08
CPK (units//L)	116.7 ± 114.5	105.3 ± 75.2	-12.1 ± 57.3	112.8 ± 75.5	119.4 ± 99.3	11.7 ± 59.4	-22.4 (-41.8, -2.99)	0.02
hs-CRP (mg/dL)	0.17 ± 0.21	0.19 ± 0.23	0.02 ± 0.23	0.13 ± 0.14	0.15 ± 0.14	0.02 ± 0.14	0.019 (-0.052, 0.090)	0.59
FPG (mg/dL)	135.7 ± 26.3	133.6 ± 31.7	-2.1 ± 26.1	128.9 ± 22.0	131.6 ± 25.1	3.0 ± 16.0	-3.8 (-12.7, 5.1)	0.40
HbA1c (%)	7.0 ± 0.6	7.3 ± 1.0	0.2 ± 0.7*	6.9 ± 0.8	7.0 ± 0.8	0.2 ± 0.4**	0.07 (-0.16, 0.29)	0.57
Total cholesterol (mg/dL)	191.84 ± 28.41	179.49 ± 31.89	-12.79 ± 23.84***	177.43 ± 35.04	172.22 ± 29.07	-4.84 ± 17.80	-3.98 (-12.3, 4.31)	0.34
CM-C (mg/dL)	2.30 ± 1.81	0.70 ± 0.67	-1.63 ± 1.78***	3.75 ± 5.03	2.53 ± 3.46	-1.37 ± 4.21*	-1.43 (-2.30, -0.57)	0.001
VLDL-C (mg/dL)	41.94 ± 15.40	27.89 ± 10.62	-14.28 ± 11.87***	38.88 ± 18.87	36.80 ± 17.55	-2.30 ± 9.62	-10.8 (-14.3, -7.3)	<0.001
LDL-C (mg/dL)	101.67 ± 20.61	101.21 ± 24.40	-0.64 ± 21.50	92.15 ± 20.80	91.37 ± 17.17	-0.29 ± 10.12	2.82 (-4.00, 9.58)	0.41
HDL-C (mg/dL)	45.94 ± 10.03	49.69 ± 10.59	3.76 ± 6.36***	42.66 ± 9.32	41.52 ± 8.77	-0.88 ± 3.96	5.17 (2.99, 7.35)	<0.001
Total triglyceride (mg/dL)	228.93 ± 118.58	138.13 ± 63.36	-93.34 ± 101.24***	229.69 ± 110.16	218.14 ± 113.68	-14.16 ± 79.55	-78.7 (-105.7, -51.7)	<0.001
CM-TG (mg/dL)	16.76 ± 13.69	5.90 ± 5.59	-11.12 ± 13.29***	24.55 ± 25.90	19.11 ± 24.50	-6.34 ± 26.32	-10.8 (-17.4, -4.37)	0.001
VLDL-TG (mg/dL)	165.06 ± 97.38	90.05 ± 50.73	-77.11 ± 83.67***	160.88 ± 78.25	155.48 ± 85.31	-6.71 ± 51.56	-68.3 (-87.9, -48.8)	<0.001
LDL-TG (mg/dL)	31.32 ± 8.90	30.67 ± 8.72	-0.76 ± 6.03	28.39 ± 8.01	28.70 ± 8.14	0.14 ± 4.05	-0.38 (-2.44, 1.68)	0.72
HDL-TG (mg/dL)	15.79 ± 5.71	11.50 ± 3.95	-4.35 ± 4.35***	15.87 ± 5.95	14.85 ± 4.90	-1.24 ± 4.03*	-3.22 (-4.52, -1.92)	<0.001
RLP-C (mg/dL)	9.9 ± 5.8	5.0 ± 3.2	-5.0 ± 4.9***	10.3 ± 8.1	8.9 ± 6.5	-1.5 ± 4.9*	-3.71 (-5.07, -2.35)	<0.001

Values are mean ± SD for continuous variables at baseline, 16 weeks, and for the change during this period. The least-square mean (95% confidence interval) is shown for the difference in this change between the groups.

*P < 0.05, **P < 0.01, ***P < 0.001: significant differences between before and after the intervention by paired T-test in each group.

The *P* values indicate the results of comparisons of changes between the Pemafibrate and EPA groups using ANCOVA adjusted for the baseline value of each corresponding variable.

### 3.3 Gene expression in PBMCs

**Figure 3:**
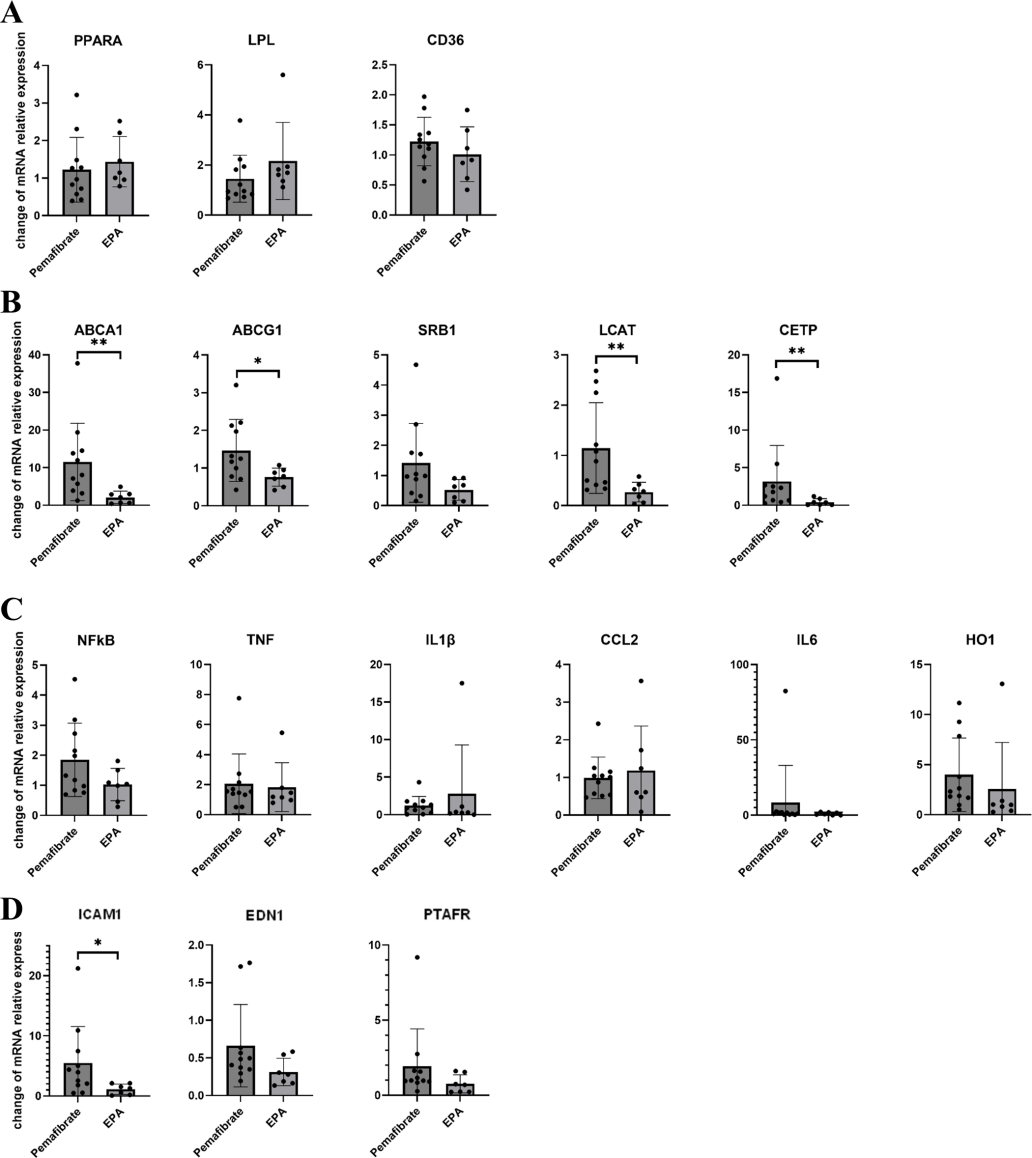
Between-group comparison of changes in PBMC gene expression after 16 weeks of treatment, categorized by gene function. **(A)** lipid metabolism-related genes (PPARA, LPL, and CD36); **(B)** cholesterol transport / reverse cholesterol transport-related genes (ABCA1, ABCGI, SCARBI, LCAT, and CETP); **(C)** inflammation/interferon / oxidative stress-related genes (NFKB1, TNFA, ILIB, CCL2, IL6, and HMOX1): **(D)** atherosclerosis / endothelial-related genes (ICAMI, EDNI, and PAFR). Changes in mRNA expression levels were compared between the pemafibrate and EPA groups in the PBMC subanalysis. Gene expression levels were normalized to HPRTI. PBMC, peripheral blood mononuclear cells; EPA, eicosapentaenoic acid. Data are presented as mean ± SD. *P < 0.05, **P < 0.01.

To assess treatment-related differences in PBMC gene expression, we compared changes in mRNA expression levels between the Pemafibrate group (n = 11) and the EPA group (n = 7) in a PBMC subgroup analysis. A *post-hoc* power/sensitivity assessment indicated that this sample size provided limited power to detect modest between-group differences. Specifically, with α = 0.05 and 80% power, the detectable standardized effect size was approximately 1.44, and this threshold increased further when accounting for multiple testing across 17 genes. Therefore, the PBMC gene-expression findings were interpreted as exploratory and used to generate hypotheses for future studies. **Figure 3** shows the between-group comparison of changes in genes related to lipid metabolism, cholesterol transport, inflammation, oxidative stress, and atherosclerosis. Several genes, including ABCA1, ABCG1, LCAT, CETP, and ICAM1, showed nominal between-group differences in expression changes. After Benjamini–Hochberg FDR adjustment, ABCA1 and LCAT remained statistically significant, whereas the other nominally significant genes did not retain significance. Raw P-values and FDR-adjusted q-values for all 17 genes are shown in **Supplementary Table 4**. Therefore, ABCA1 and LCAT were considered the most robust exploratory PBMC signals, whereas the remaining gene-expression findings were interpreted as nominal and hypothesis-generating. **Supplementary Figure 2** shows the within-group changes in PBMC gene expression. Baseline characteristics of participants included in and excluded from the PBMC gene-expression substudy are shown in **Supplementary Table 5**. Participants included in the PBMC substudy had lower baseline MDA-LDL levels and more frequent use of GLP-1 receptor agonists than those not included. These differences suggest that non-random selection related to RNA quantity or quality cannot be fully excluded. Accordingly, the PBMC findings should be interpreted cautiously and should not be generalized to the entire study population without validation.

### 3.4 Relationship among MDA-LDL, small dense LDL, and gene expression

No significant direct correlation was observed between the change in MDA-LDL and the change in any individual gene expression level in either group. Correlation coefficients and P-values for the associations between changes in MDA-LDL and changes in each PBMC gene-expression marker are summarized in **Supplementary Table 6**. Representative scatter plots are also provided in **Supplementary Figure 3**. In contrast, the Pemafibrate group showed a significant reduction in small dense LDL, and the change in small dense LDL was positively correlated with the change in MDA-LDL (**Supplementary Figure 4**). We further explored the relationships among lipid-related markers, PBMC gene expression, and MDA-LDL using structural equation modeling (SEM) (**Supplementary Figure 5**). The hypothesized models showed only poor fit in both groups and were therefore considered exploratory. The model fit indices were poor in both treatment groups, including CFI values of 0.347 in the Pemafibrate group and 0.237 in the EPA group, indicating that the models did not adequately fit the observed data structure. Therefore, the SEM findings were used as exploratory visualizations to support hypothesis generation rather than mechanistic inference. Nevertheless, these exploratory models may help generate hypotheses regarding potential relationships among lipid remodeling, PBMC gene-expression changes, and MDA-LDL reduction.

## 4 Discussion

In this prespecified subanalysis of the PARADISE in Kagoshima study, we identified two main findings. First, pemafibrate was associated with a greater 16-week reduction in MDA-LDL than EPA in patients with type 2 diabetes and hypertriglyceridemia. Second, the reduction in MDA-LDL was associated with a reduction in small dense LDL, particularly in the Pemafibrate group. We further explored the PBMC gene expression changes and found that pemafibrate and EPA were associated with different patterns of gene changes, with ABCA1 and LCAT remaining significant after FDR adjustment.

The greater reduction in MDA-LDL with pemafibrate is a notable finding of this study. Previous reports have shown that fibrates such as bezafibrate and fenofibrate can reduce MDA-LDL or related oxidized LDL parameters in patients with dyslipidemia and type 2 diabetes (11, 13). In contrast, the MDA-LDL-lowering effect of pemafibrate has not been well documented. To our knowledge, this is the first study to demonstrate an association between pemafibrate treatment and greater MDA-LDL reduction compared with EPA in patients with type 2 diabetes and hypertriglyceridemia. Our findings therefore extend the known lipid-modifying effects of pemafibrate beyond triglyceride lowering and suggest that pemafibrate may also favorably influence oxidized LDL-related markers. However, the clinical relevance of this magnitude of MDA-LDL reduction remains uncertain. Although MDA-LDL has been associated with atherosclerotic burden and cardiovascular risk in observational studies, it has not been established as a validated therapeutic surrogate. Thus, whether drug-induced reductions in MDA-LDL directly lead to reductions in cardiovascular events remains unknown. At the same time, urinary 8-OHdG did not change significantly in either group (**Figure 2**), but this finding should be interpreted cautiously. Urinary 8-OHdG primarily reflects oxidative DNA damage and is not a lipid-specific marker of oxidative modification. Therefore, the absence of a parallel change in urinary 8-OHdG does not necessarily indicate that systemic lipid peroxidation was unaffected. More direct lipid peroxidation markers, such as plasma isoprostanes or lipid peroxidation-derived aldehydes (e.g., 4-hydroxy-nonenal), would have provided a more appropriate comparison with MDA-LDL and should be considered in future studies.

This point is clinically relevant in light of the differing cardiovascular outcome data for triglyceride-lowering therapies. EPA-based therapy has been shown to reduce cardiovascular events in large-scale outcome trials, whereas pemafibrate did not reduce cardiovascular events in the PROMINENT trial despite marked triglyceride lowering. Importantly, all participants in PROMINENT were receiving statin therapy. These outcome data emphasize that biomarker changes should not be equated with clinical benefit. In the present study, pemafibrate was associated with greater reductions in small dense LDL, RLP-C, and MDA-LDL over 16 weeks than EPA. These findings suggest that pemafibrate and EPA may differ in their effects on selected lipid-quality biomarkers. However, the greater MDA-LDL reduction observed with pemafibrate should not be interpreted as clinical superiority over EPA, because EPA has demonstrated cardiovascular outcome benefit whereas pemafibrate has not. Whether such differences in biomarker profiles translate into vascular or clinical outcomes cannot be determined from the present study. The observed between-group difference in MDA-LDL (~21 U/L) may be interpreted in context because previous observational studies have reported differences in circulating MDA-LDL levels between individuals with and without coronary artery disease of a broadly comparable order (11). In addition, experimental studies in mice have suggested that immune responses to MDA-LDL-related epitopes may influence atherosclerosis; for example, MDA-LDL immunization has been shown to induce germinal-center-derived anti-MDA-LDL antibody responses with atheroprotective features. These findings support the concept that MDA-LDL may represent not only a biomarker of oxidative lipoprotein modification but also a biologically relevant factor in atherosclerotic processes (19). However, such comparisons are indirect and should not be used to infer that the present treatment-associated change would translate into a reduction in atherosclerotic progression or cardiovascular events. More broadly, cardiometabolic risk management in patients with diabetes and coronary artery disease requires integration of lipid abnormalities, glycemic control, inflammation, vascular function, renal protection, and weight-related metabolic risk. In this broader context, the present findings provide hypothesis-generating evidence regarding pemafibrate-associated changes in lipid-quality biomarkers, but they do not establish cardiovascular benefit.

The relationship between small dense LDL and MDA-LDL is particularly important. Small dense LDL particles are more susceptible to oxidative modification and are considered highly atherogenic. In the present study, the change in small dense LDL was positively correlated with the change in MDA-LDL (**Supplementary Figure 4**), suggesting that improvement in LDL particle quality may be an important determinant of MDA-LDL reduction. However, this association should be interpreted as hypothesis-generating because of the limited sample size and exploratory nature of the analysis. In PROMINENT, all participants were receiving statin therapy and had relatively well-controlled LDL-C levels at baseline. In contrast, approximately half of the participants in the present cohort were receiving anti-hyperlipidemic therapy. Because statins can reduce oxidized LDL-related markers, modulate lipoprotein particle profiles, and affect cholesterol transport-related gene expression, background statin use may have influenced the present findings. A sensitivity analysis stratified by baseline statin use is provided in **Supplementary Figure 1**. In this descriptive analysis, the reduction in MDA-LDL with pemafibrate appeared numerically greater among statin users. However, because of the limited sample size and the absence of a formal treatment-by-statin interaction test, baseline statin use cannot be considered a confirmed effect modifier. Residual confounding or effect modification by statin therapy therefore cannot be excluded. This view is also supported by prior fenofibrate studies showing improvement in small dense LDL and remnant-rich lipoprotein fractions, together with a reduction in MDA-LDL, suggesting that modulation of lipoprotein quality may be an important component of fibrate-class effects beyond absolute triglyceride lowering (14). SEM was performed as an exploratory visualization of potential multivariable relationships among triglyceride reduction, small dense LDL, PBMC gene-expression changes, and MDA-LDL. The hypothesized models showed poor fit in both treatment groups, indicating that the proposed path structure did not adequately explain the observed covariance structure. Therefore, the SEM findings should not be interpreted as evidence of a confirmed mechanistic pathway. Nevertheless, the poor fit itself may be informative, suggesting that the relationships among triglyceride reduction, small dense LDL remodeling, PBMC gene-expression changes, and MDA-LDL are more complex than assumed in the prespecified model. Within this limitation, the observed patterns raise the hypothesis that pemafibrate and EPA may be associated with MDA-LDL reduction through potentially different biological pathways. In particular, the pemafibrate-associated MDA-LDL reduction may be related to triglyceride-rich lipoprotein remodeling, small dense LDL reduction, and PBMC gene-expression changes related to cholesterol handling and reverse cholesterol transport, whereas EPA-associated effects may involve mechanisms less directly captured by the present model.

Another finding of this study is the difference in PBMC gene expression between pemafibrate and EPA. After FDR adjustment, ABCA1 and LCAT remained statistically significant, whereas other genes showed only nominal differences. Because ABCA1 and LCAT are involved in cholesterol handling and reverse cholesterol transport, these findings may reflect exploratory PBMC signals related to cholesterol handling pathways. However, PBMC gene expression should not be regarded as a substitute for hepatic or vascular tissue responses, and the small RNA subsample limits mechanistic interpretation.

Importantly, no significant direct correlation was observed between the change in MDA-LDL and the change in any individual gene-expression marker. Therefore, the PBMC findings should not be interpreted as evidence that transcriptional changes directly mediated MDA-LDL reduction. Rather, together with the exploratory SEM results, these findings raise the hypothesis that pemafibrate and EPA may differ not only in their quantitative lipid effects but also in their relationships with cholesterol-handling responses in PBMCs. This interpretation remains hypothesis-generating and requires validation in larger studies.

More broadly, cardiometabolic risk management in patients with diabetes and coronary artery disease increasingly requires integration of lipid abnormalities, glycemic control, inflammation, vascular function, renal protection, and weight-related metabolic risk. A recent scoping review highlighted the evolving therapeutic landscape of metabolic risk management in coronary artery disease, including SGLT2 inhibitors, GLP-1 receptor agonists, pemafibrate, and other emerging strategies (20). In this broader context, the present findings may provide hypothesis-generating evidence regarding pemafibrate-associated changes in lipid-quality biomarkers, but they do not establish cardiovascular benefit. Our data suggest that the biological consequences of triglyceride lowering may need to be considered at multiple levels: the primary metabolic effects in the liver and lipoprotein lipase system, and peripheral cellular responses related to monocyte/macrophage-lineage cholesterol efflux, as potentially reflected in PBMC gene-expression changes. The present findings therefore raise the possibility that treatment-related differences in lipoprotein quality and peripheral cellular responses, rather than triglyceride lowering alone, may be relevant to understanding why marked triglyceride reduction does not uniformly translate into cardiovascular benefit (17, 20).

Several limitations should be acknowledged. First, this was a prespecified subanalysis of an open-label randomized trial, and the sample size was limited for several exploratory outcomes. Although MDA-LDL, lipoprotein fractions, and PBMC gene expression were objective laboratory outcomes, because the trial was open-label, treatment awareness may have influenced adherence, lifestyle behaviors, and concomitant medication use, which could have affected lipid and inflammatory biomarkers. Second, the PBMC gene-expression analysis was performed in a small subset of participants from whom sufficient RNA quantity could be obtained. Participants included in the PBMC substudy differed from those not included in some baseline characteristics, including lower MDA-LDL levels and more frequent GLP-1 receptor agonist use. Therefore, non-random selection related to RNA availability cannot be excluded. Third, the PBMC substudy was underpowered to detect small-to-moderate between-group differences, and multiple genes were tested; therefore, nominally significant gene-expression findings may include false-positive results. FDR-adjusted q-values are provided, and these findings should be interpreted as exploratory. Fourth, PBMC gene expression cannot be considered a direct surrogate for hepatic lipid metabolism. Fifth, the SEM analyses showed poor model fit and therefore do not support definitive mechanistic inference. Sixth, baseline statin use and other anti-hyperlipidemic medications may have influenced MDA-LDL, lipoprotein fractions, and gene-expression profiles, and residual confounding or effect modification cannot be excluded. Finally, although pemafibrate was associated with a reduction in MDA-LDL, MDA-LDL has not been validated as a therapeutic surrogate for cardiovascular event reduction. Larger studies incorporating vascular imaging or clinical endpoints are needed to determine the clinical significance of these biomarker changes.

## 5 Conclusion

In this prespecified subanalysis of the PARADISE in Kagoshima study, pemafibrate was associated with greater 16-week reductions in MDA-LDL and small dense LDL fractions than EPA in patients with type 2 diabetes and hypertriglyceridemia. These findings suggest that pemafibrate may modify selected biomarkers of lipoprotein quality. However, the clinical relevance of this MDA-LDL reduction, including its implications for atherosclerotic progression or cardiovascular event reduction, remains unknown. Exploratory PBMC gene-expression analyses suggested possible changes in lipid transport- and inflammation-related pathways, particularly involving ABCA1 and LCAT, but these findings should be interpreted cautiously because of the small RNA subsample, limited statistical power, and multiple testing.

## Data Availability

The data that support the findings of this study are available from the corresponding author upon reasonable request.

## References

[1] Ginsberg HN Zhang YL Hernandez-Ono A . Regulation of plasma triglycerides in insulin resistance and diabetes. Arch Med Res. (2005) 36:232–40. doi: 10.1016/j.arcmed.2005.01.005 15925013

[2] Ye X Kong W Zafar MI Chen LL . Serum triglycerides as a risk factor for cardiovascular diseases in type 2 diabetes mellitus: a systematic review and meta-analysis of prospective studies. Cardiovasc Diabetol. (2019) 18:48. doi: 10.1186/s12933-019-0851-z 30987625 PMC6466658

[3] Kristensen FPB Christensen DH Mortensen MB Maeng M Kahlert J Toft H . Triglycerides and risk of cardiovascular events in statin-treated patients with newly diagnosed type 2 diabetes: a Danish cohort study. Cardiovasc Diabetol. (2023) 22:173. doi: 10.1186/s12933-023-01921-5 37495999 PMC10373341

[4] Ginsberg HN Packard CJ Chapman MJ Borén J Aguilar-Salinas CA Averna M . Triglyceride-rich lipoproteins and their remnants: metabolic insights, role in atherosclerotic cardiovascular disease, and emerging therapeutic strategies-a consensus statement from the European Atherosclerosis Society. Eur Heart J. (2021) 42:4791–806. doi: 10.1093/eurheartj/ehab551 34472586 PMC8670783

[5] Wadström BN Pedersen KM Wulff AB Nordestgaard BG . Elevated remnant cholesterol, plasma triglycerides, and cardiovascular and non-cardiovascular mortality. Eur Heart J. (2023) 44(16):1432–45:1432–45. doi: 10.1093/eurheartj/ehac822 36631967

[6] Ohmura H . Contribution of remnant cholesterol to coronary atherosclerosis. J Atheroscler Thromb. (2022) 29:1706–8. doi: 10.5551/jat.ed205 35691847 PMC9881543

[7] Tian L Long S Li C Liu Y Chen Y Zeng Z . High-density lipoprotein subclass and particle size in coronary heart disease patients with or without diabetes. Lipids Health Dis. (2012) 11:54. doi: 10.1186/1476-511x-11-54 22584085 PMC3477075

[8] Ishibashi S Yamashita S Arai H Araki E Yokote K Suganami H . Effects of K-877, a novel selective PPARα modulator (SPPARMα), in dyslipidaemic patients: a randomized, double-blind, active- and placebo-controlled, phase 2 trial. Atherosclerosis. (2016) 249:36–43. doi: 10.1016/j.atherosclerosis.2016.02.029 27062408

[9] Lankin VZ Tikhaze AK Melkumyants AM . Malondialdehyde as an important key factor of molecular mechanisms of vascular wall damage under heart diseases development. Int J Mol Sci. (2022) 24:128. doi: 10.3390/ijms24010128 36613568 PMC9820205

[10] Virella G Wilson K Elkes J Hammad S Rajab H Li Y . Immune complexes containing malondialdehyde (MDA) LDL induce apoptosis in human macrophages. Clin Immunol. (2018) 187:1–9. doi: 10.1016/j.clim.2017.06.010 28689783

[11] Tajika K Okamatsu K Takano M Inami S Yamamoto M Murakami D . Malondialdehyde-modified low-density lipoprotein is a useful marker to identify patients with vulnerable plaque. Circ J. (2012) 76:2211–7. doi: 10.1253/circj.cj-12-0183 22785057

[12] Tanaga K Bujo H Inoue M Mikami K Kotani K Takahashi K . Increased circulating malondialdehyde-modified LDL levels in patients with coronary artery diseases and their association with peak sizes of LDL particles. Arterioscler Thromb Vasc Biol. (2002) 22:662–6. doi: 10.1161/01.atv.0000012351.63938.84 11950707

[13] Kondo A Morita H Nakamura H Kotani K Kobori K Ito S . Influence of fibrate treatment on malondialdehyde-modified LDL concentration. Clin Chim Acta. (2004) 339:97–103. doi: 10.1016/j.cccn.2003.09.005 14687899

[14] Shinnakasu A Yamamoto K Kurano M Arimura H Arimura A Kikuti A . The combination therapy of fenofibrate and ezetimibe improved lipid profile and vascular function compared with statins in patients with type 2 diabetes. J Atheroscler Thromb. (2017) 24:735–48. doi: 10.5551/jat.39446 28450679 PMC5517547

[15] Yokoyama M Origasa H Matsuzaki M Matsuzawa Y Saito Y Ishikawa Y . Effects of eicosapentaenoic acid on major coronary events in hypercholesterolaemic patients (JELIS): a randomized open-label, blinded endpoint analysis. Lancet. (2007) 369:1090–8. doi: 10.1016/s0140-6736(07)60527-3 17398308

[16] Bhatt DL Steg PG Miller M Brinton EA Iacobson TA Ketchum SB . Cardiovascular risk reduction with icosapent ethyl for hypertriglyceridemia. N Engl J Med. (2019) 380:11–22. doi: 10.1056/nejmoa1812792 30415628

[17] Pradhan AD Glynn RJ Fruchart JC MacFadyen JG Zaharris ES Everett BM . Triglyceride lowering with pemafibrate to reduce cardiovascular risk. N Engl J Med. (2022) 387:1923–34. doi: 10.1056/nejmoa2210645 36342113

[18] Sasaki Y Raza-Iqbal S Tanaka T Murakami K Anai M Osawa T . Gene expression profiles induced by a novel selective peroxisome proliferator-activated receptor α modulator (SPPARMα) pemafibrate. Int J Mol Sci. (2019) 20:5682. doi: 10.3390/ijms20225682 31766193 PMC6888257

[19] Martos-Folgado I del Monte-Monge A Lorenzo C Busse CE Delgado P Mur S . MDA-LDL vaccination induces athero-protective germinal-center-derived antibody responses. Cell Rep. (2022) 41:1–14. doi: 10.1016/j.celrep.2022.111468 36223741

[20] Gudla SS Bhumireddy SKA Vadaga AK Nandula MS . Managing metabolic risks in coronary artery disease: A scoping review of recent clinical and therapeutic evidence. J Diabetes Metab Disord. (2026) 25:118. doi: 10.1007/s40200-026-01916-5 41867418 PMC13003029

