# Supplementary material for: Pemafibrate is associated with greater reductions in MDA-LDL and small dense LDL fractions with exploratory PBMC gene-expression changes: a prespecified subanalysis of the PARADISE in Kagoshima study
Source: Front Clin Diabetes Healthc. 2026 Jul 1;7:1850470. doi: 10.3389/fcdhc.2026.1850470 (PMC13368630; doi:10.3389/fcdhc.2026.1850470)
Supplement: Supplementary file 1 [file DataSheet1.pdf]

Supplementary Table S1.

TaqMan Primers and probe (TaqMan Gene Expression Assays, Applied Biosystems) and Assay ID

Target gene	Assay ID	Accession #	Amplicon length (bp)
NFkB1	Hs00765730_m1	NM_1165412	66
TNFa	Hs00174128_m1	NM_000594	80
IL-1b	Hs01555410_m1	NM_000576	91
CCL2	Hs00234140_m1	NM_002982	101
IL-6	Hs00174131_m1	NM_000600	95
HO1	Hs01110250_m1	NM_002133	82
LPL	Hs00173425_m1	NM_000237	103
ABCA1	Hs01059101_m1	NM_005502	63
ABCG1	Hs00245154_m1	NM_004915	58
SR-B1	Hs00969821_m1	NM_001082959	73
LCAT	Hs01068069_m1	NM_000229	84
CETP	Hs00163942_m1	NM_000078	67
PPARA	Hs00947536_m1	NM_001001928	62
CD36	Hs00354519_m1	NM_000072	83
EDN1	Hs00174961_m1	NM_001168319	62
ICAM1	Hs00164932_m1	NM_000201	87
PAFR	Hs00265399_s1	NM_000952	98
HPRT1	Hs02800695_m1	NM_000194	82

NFkB1, nuclear factor kappa B subunit 1; TNFa, tumor necrosis factor a; IL-1b, interleukin 1b; CCL2, C-C motif chemokine ligand 2; IL-6, interleukin 6; HO1, heme oxygenase 1; LPL, lipoprotein lipase; ABCA1, ATP binding cassette subfamily A member 1; ABCG1, binding cassette subfamily G member 1; SR-B1, scavenger recepter class B member 1; LCAT, lecithin-cholesterol acyltransferase; CETP, cholesteryl ester transfer protein; PPARA, peroxisome proliferator activated receptor alpha; CD36, cluster differentiation 36; EDN1, endothelin 1; ICAM1, intercellular adhesion molecule 1; PAFR, platelet activating factor receptor; HPRT1, Hypoxanthine phosphoribosyltransferase1.

Supplementary Table S2. Assessment of ANCOVA assumptions for primary and secondary outcomes.

	homogeneity of slopes	linearity of responses
Body Weight (kg)	○	×
AST (units/L)	○	○
ALT (units/L)	○	○
γGT (units/L)	○	○
sCr (mg/dL)	○	×
CPK (units/L)	×	○
hs-CRP (mg/dL)	○	○
FPG (mg/dL)	○	○
HbA1c (%)	○	×
Total cholesterol (mg/dL)	○	○
CM-C (mg/dL)	○	○
VLDL-C (mg/dL)	×	○
LDL-C (mg/dL)	○	○
HDL-C (mg/dL)	○	○
Total triglyceride (mg/dL)	×	○
CM-TG (mg/dL)	○	○
VLDL-TG (mg/dL)	×	○
LDL-TG (mg/dL)	○	○
HDL-TG (mg/dL)	○	○
RLP-C (mg/dL)	×	○

Assumptions for ANCOVA, including homogeneity of regression slopes and linearity of responses, were assessed for each variable.

Supplementary Table S3. Within-group changes and between-group comparisons of change scores (complementary analysis)

	Pemafibrate group (n=51)			EPA group (n=46)			*P* value
	Baseline	Week 16	Change	Baseline	Week 16	Change	
Body Weight (kg)	75.4±19.8	75.2±19.5	-0.2±1.8	77.9±17.0	78.7±17.4	0.3±1.6	0.20
AST (units/L)	28.1±13.6	26.5±13.5	-1.2±12.1	26.0±9.2	31.2±16.7	5±16	0.04
ALT (units/L)	35.2±21.6	29.4±22.6	-5.8±16.4*	32.5±17.2	41.2±41.5	7.9±42	0.03
γGT (units/L)	44.0±32.8	29.2±25.5	-14.7±15.2***	53.2±37.8	57.4±43.2	3.2±29	<0.001
sCr (mg/dL)	0.77±0.20	0.79±0.21	0.02±0.07	0.78±0.20	0.77±0.20	0.02±0.06	0.07
CPK (units//L)	116.7±114.5	105.3±75.2	-12.1±57.3	112.8±75.5	119.4±99.3	11.7±59.4	0.052
hs-CRP (mg/dL)	0.17±0.21	0.19±0.23	0.02±0.23	0.13±0.14	0.15±0.14	0.02±0.14	0.89
FPG (mg/dL)	135.7±26.3	133.6±31.7	-2.1±26.1	128.9±22.0	131.6±25.1	3.0±16.0	0.26
HbA1c (%)	7.0±0.6	7.3±1.0	0.2±0.7*	6.9±0.8	7.0±0.8	0.2±0.4**	0.51
Total cholesterol (mg/dL)	191.84±28.41	179.49±31.89	-12.79±23.84 ***	177.43±35.04	172.22±29.07	-4.84±17.80	0.07
CM-C (mg/dL)	2.30±1.81	0.70±0.67	-1.63±1.78***	3.75±5.03	2.53±3.46	-1.37±4.21*	0.70
VLDL-C (mg/dL)	41.94±15.40	27.89±10.62	-14.28±11.87***	38.88±18.87	36.80±17.55	-2.30±9.62	<0.001
LDL-C (mg/dL)	101.67±20.61	101.21±24.40	-0.64±21.50	92.15±20.80	91.37±17.17	-0.29±10.12	0.92
HDL-C (mg/dL)	45.94±10.03	49.69±10.59	3.76±6.36***	42.66±9.32	41.52±8.77	-0.88±3.96	<0.001
Total triglyceride (mg/dL)	228.93±118.58	138.13±63.36	-93.34±101.24***	229.69±110.16	218.14±113.68	-14.16±79.55	<0.001
CM-TG (mg/dL)	16.76±13.69	5.90±5.59	-11.12±13.29***	24.55±25.90	19.11±24.50	-6.34±26.32	0.26
VLDL-TG (mg/dL)	165.06±97.38	90.05±50.73	-77.11±83.67***	160.88±78.25	155.48±85.31	-6.71±51.56	<0.001
LDL-TG (mg/dL)	31.32±8.90	30.67±8.72	-0.76±6.03	28.39±8.01	28.70±8.14	0.14±4.05	0.40
HDL-TG (mg/dL)	15.79±5.71	11.50±3.95	-4.35±4.35***	15.87±5.95	14.85±4.90	-1.24±4.03*	<0.001
RLP-C (mg/dL)	9.9±5.8	5.0±3.2	-5.0±4.9***	10.3±8.1	8.9±6.5	-1.5±4.9*	<0.001

Values are mean ± SD at baseline, week 16, and for changes.

* *P* <0.05, ** *P* <0.01, *** *P* <0.001: Within-group comparisons were performed using paired t-tests.

Between-group comparisons of change scores were performed using unpaired t-tests. Values are mean ± SD at baseline, week 16, and for changes.

These analyses were performed as complementary analyses to assess the robustness of the primary ANCOVA results.

Supplementary Table S4. Between-group comparisons of changes in PBMC gene-expression markers with raw P values and FDR-adjusted q values.

Gene	Change in Pemafibrate group (n=11)	Change in EPA group (n=7)	difference	Cohen's d	Raw P value (unpaired t-test)	FDR-adjusted q value (BH)	FDR q <0.05	Welch P value (sensitivity)	Mann-Whitney P value (sensitivity)
PPARA	0±0.954	-0.401±0.635	0.401	0.473	0.342	0.388	No	0.300	0.425
LPL	-0.321±0.792	-0.908±0.745	0.587	0.758	0.137	0.211	No	0.135	0.179
ABCA1	-3.006±1.362	-0.578±1.37	-2.429	-1.779	0.002	0.029	Yes	0.003	0.002
EDN1	0.939±0.98	1.882±0.833	-0.943	-1.016	0.052	0.145	No	0.046	0.069
ABCG1	-0.341±0.849	0.464±0.502	-0.805	-1.090	0.039	0.131	No	0.023	0.044
LCAT	0.227±1.171	2.424±1.54	-2.197	-1.662	0.003	0.029	Yes	0.009	0.006
CETP	-0.656±1.735	2.08±1.857	-2.736	-1.536	0.006	0.033	Yes	0.009	0.008
SCARB1	0.049±1.409	1.342±1.296	-1.293	-0.945	0.068	0.145	No	0.066	0.035
CD36	-0.213±0.507	0.119±0.7	-0.332	-0.567	0.258	0.338	No	0.302	0.328
IL1B	-0.169±2.437	-1.469±2.388	1.300	0.537	0.283	0.343	No	0.284	0.104
PAFR	-0.354±1.246	0.857±1.276	-1.211	-0.964	0.064	0.145	No	0.070	0.056
ICAM1	-1.678±1.681	0.57±1.904	-2.248	-1.272	0.018	0.077	No	0.026	0.027
NFKB1	-0.617±0.915	0.189±0.951	-0.807	-0.869	0.091	0.159	No	0.099	0.211
CCL2	0.186±0.714	0.448±1.707	-0.262	-0.221	0.654	0.695	No	0.711	1.000
TNF	-0.641±1.069	-0.561±0.914	-0.080	-0.079	0.872	0.872	No	0.867	0.536
IL6	-0.484±2.219	1.106±2.393	-1.590	-0.696	0.169	0.240	No	0.183	0.375
HMOX1 / HO-1	-1.427±1.381	-0.088±1.801	-1.339	-0.863	0.093	0.159	No	0.123	0.069

Change indicates week 16 minus baseline expression. Raw P values are from unpaired t-tests. FDR-adjusted q values were calculated using the Benjamini–Hochberg method across 17 genes.

Supplementary Table S5. Baseline characteristics of participants included in and excluded from the PBMC gene-expression substudy.

	PBMC included (n=18)	PBMC excluded (n=79)	P-value
Treatment group, pemafibrate	11 (61.1)	40 (50.6)	0.447
Age, years	53.0 ± 9.7	58.0 ± 10.7	0.071
Male sex	12 (66.7)	47 (59.5)	0.790
Body mass index, kg/m^2	30.1 ± 6.7	28.1 ± 5.6	0.199
Systolic BP, mmHg	126.3 ± 12.8	129.3 ± 14.2	0.409
Diastolic BP, mmHg	83.9 ± 9.2	79.5 ± 11.2	0.129
HbA1c, %	6.7 ± 0.6	7.0 ± 0.7	0.147
Fasting plasma glucose, mg/dL	124.7 ± 18.5	134.3 ± 25.4	0.136
eGFR, mL/min/1.73 m^2	74.0 ± 15.2	77.5 ± 20.5	0.505
Triglycerides, mg/dL	254.2 ± 126.4	212.4 ± 109.2	0.157
LDL-C, mg/dL	110.1 ± 23.1	115.6 ± 26.2	0.413
HDL-C, mg/dL	44.9 ± 8.6	48.2 ± 11.1	0.242
RLP-C, mg/dL	11.3 ± 6.7	9.8 ± 7.0	0.430
MDA-LDL, U/L	109.0 ± 25.0	138.7 ± 41.9	0.005
Anti-hyperlipidemic medication	7 (38.9)	38 (48.1)	0.603
Statin use	5 (27.8)	30 (38.0)	0.588
SGLT2 inhibitor use	9 (50.0)	37 (46.8)	1.000
GLP-1 receptor agonist use	6 (33.3)	10 (12.7)	0.071
DPP-4 inhibitor use	10 (55.6)	46 (58.2)	1.000
Biguanide use	17 (94.4)	69 (87.3)	0.683
Current smoking	7 (38.9)	19 (24.1)	0.241

Continuous variables are mean ± SD and compared using unpaired t-tests. Categorical variables are n (%) and compared using Fisher exact tests. P values are exploratory.

Supplementary Table S6. Correlations between changes in MDA-LDL and changes in PBMC gene-expression markers.

Target gene (n=18)	Correlation coefficient	P value
PPARA	-0.081	0.751
LPL	0.025	0.922
ABCA1	0.396	0.104
EDN1	0.105	0.677
ABCG1	0.033	0.896
LCAT	0.225	0.369
CETP	0.116	0.648
SCARB1	-0.117	0.645
CD36	0.254	0.309
IL1B	-0.209	0.406
PAFR (PTAFR)	-0.188	0.455
ICAM1	0.318	0.198
NFKB1	-0.036	0.887
CCL2	-0.094	0.711
TNF	0.134	0.595
IL6	-0.068	0.788
HMOX1 / HO-1	0.191	0.447

Correlations were calculated in the PBMC gene-expression substudy using the Spearman method.

NFkB1, nuclear factor kappa B subunit 1; TNFa, tumor necrosis factor a; IL-1b, interleukin 1b; CCL2, C-C motif chemokine ligand 2; IL-6, interleukin 6; HO1, heme oxygenase 1; LPL, lipoprotein lipase; ABCA1, ATP binding cassette subfamily A member 1; ABCG1, binding cassette subfamily G member 1; SR-B1, scavenger recepter class B member 1; LCAT, lecithin-cholesterol acyltransferase; CETP, cholesteryl ester transfer protein; PPARA, peroxisome proliferator activated receptor alpha; CD36, cluster differentiation 36; EDN1, endothelin 1; ICAM1, intercellular adhesion molecule 1; PAFR, platelet activating factor receptor.

Supplementary Figure S1. Sensitivity analyses stratified by baseline statin use for changes in MDA-LDL.

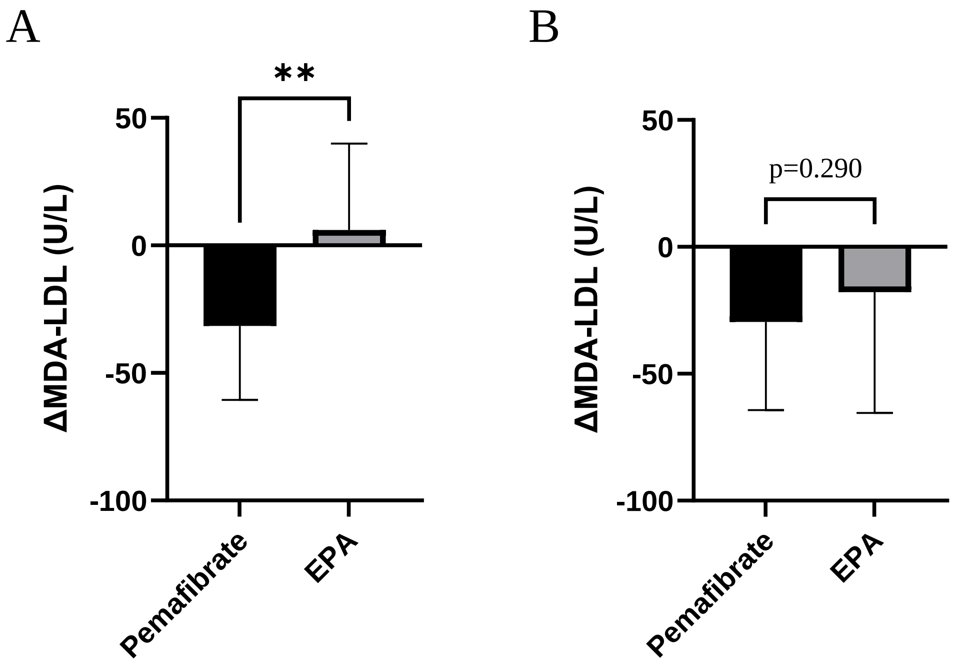

Supplementary Figure S1. Sensitivity analyses stratified by baseline statin use for changes in MDA-LDL.
Changes in MDA-LDL from baseline to week 16 are shown in the pemafibrate and EPA groups, stratified by baseline statin use. Data are presented as mean ± SD. **A**: Participants receiving statins at baseline. **B**: Participants not receiving statins at baseline. P values were calculated using the unpaired t test with Welch's correction. These analyses were descriptive and exploratory, and formal treatment-by-statin interaction tests were not performed. ** *P* <0.01.

Supplementary Figure S2.

A

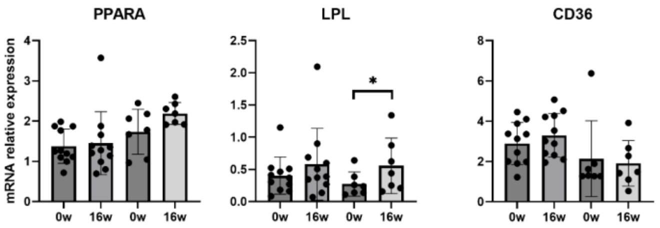

B

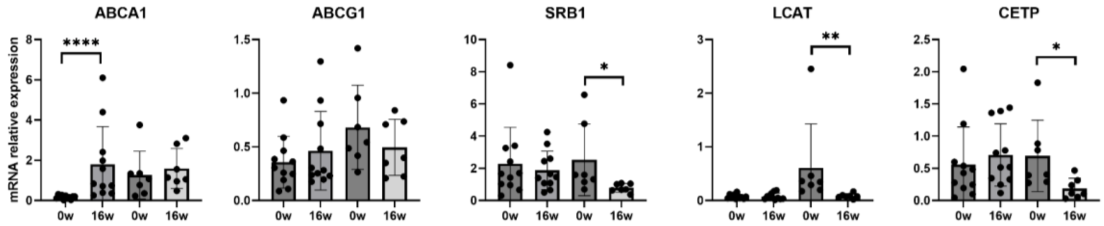

C

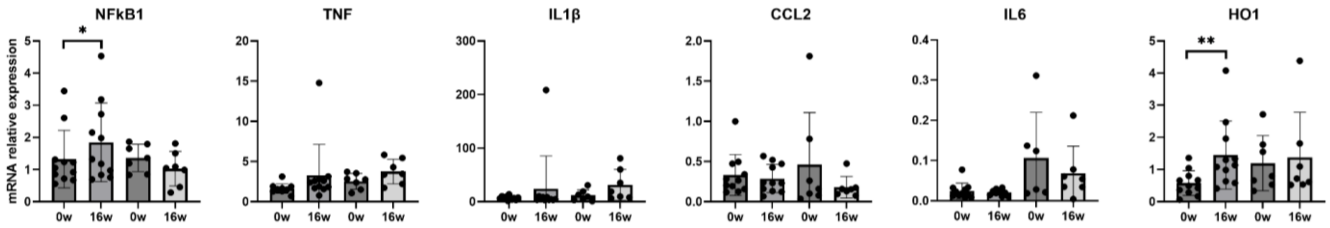

D

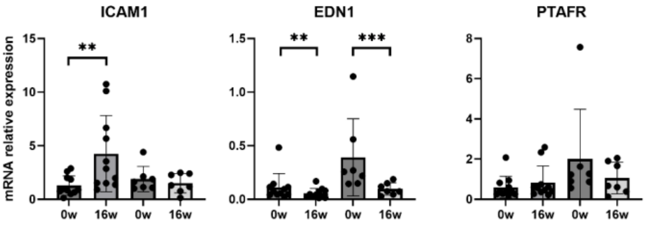

Supplementary Figure S2. Within-group changes in PBMC gene expression after 16 weeks of treatment, categorized by gene function.

A: lipid metabolism-related genes (PPARA, LPL, and CD36); **B:** cholesterol transport / reverse cholesterol transport-related genes (ABCA1, ABCG1, SCARB1, LCAT, and CETP); **C:** inflammation / interferon / oxidative stress-related genes (NFKB1, TNFA, IL1B, CCL2, IL6, HMOX1, and IFIT1B); **D:** atherosclerosis / endothelial-related genes (ICAM1, EDN1, and PAFR). Baseline (week 0) and week 16 values are shown separately for the pemafibrate and EPA groups. P values were obtained using the paired t-test for within-group comparisons. Gene expression levels were normalized to HPRT1. PBMC, peripheral blood mononuclear cells; EPA, eicosapentaenoic acid.

Supplementary Figure S3. Relationship between change of MDA-LDL and change of mRNA relative expression.

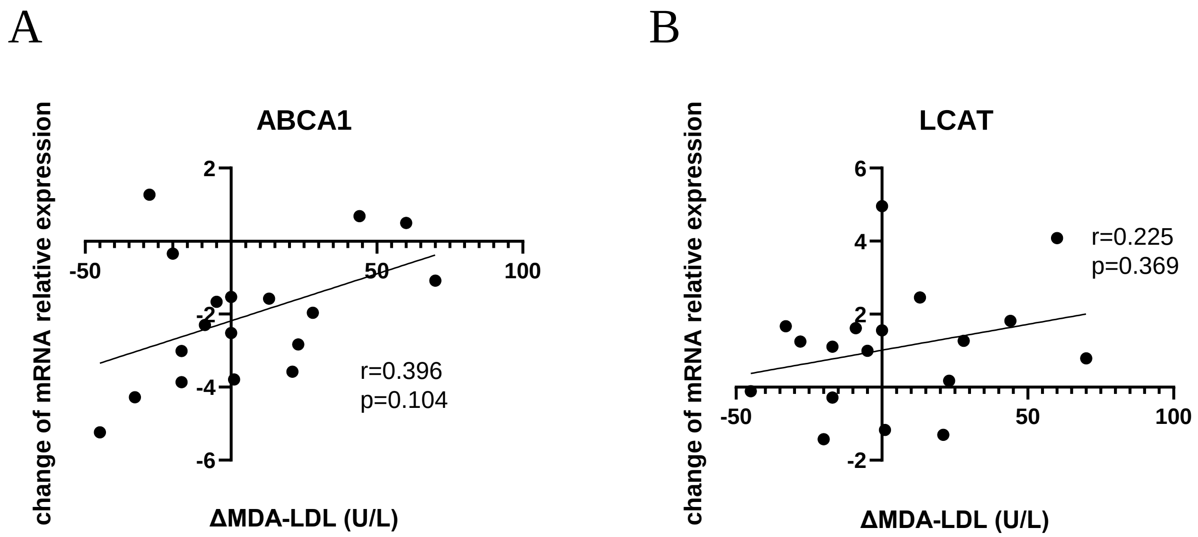

Supplementary Figure S3. Relationship between changes in MDA-LDL and changes in relative mRNA expression.

The relative expression of each gene was calculated using the 2^-ΔCt method based on real-time RT-PCR data. The correlation coefficient was calculated using Spearman's rank correlation method.

A: Change in ABCA1 mRNA expression and change in serum MDA-LDL.

B: Change in LCAT mRNA expression and change in serum MDA-LDL.

Supplementary Figure S4. Relationship between change of MDA-LDL and small dense LDL in the Pemafibrate group

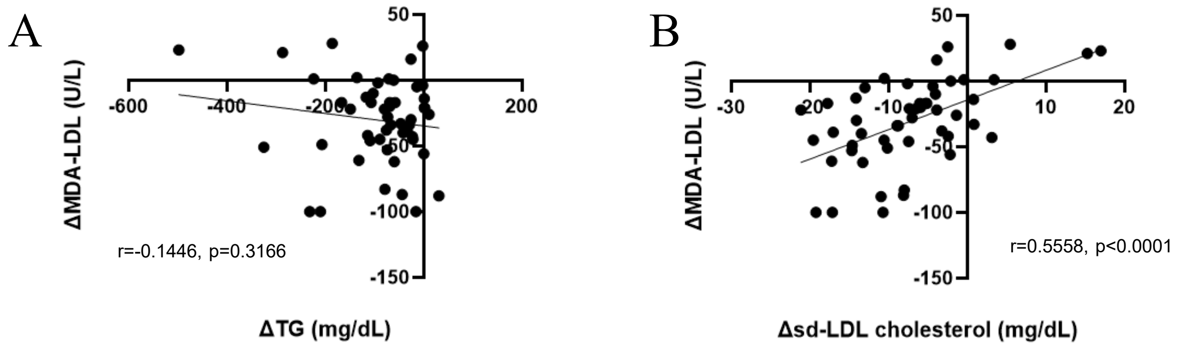

Supplementary Figure S4. Correlation analyses of changes in MDA-LDL in the pemafibrate group.
A: Scatter plot with regression line showing the relationship between the change in MDA-LDL and the change in TG in the pemafibrate group. **B:** Scatter plot with regression line showing the relationship between the change in MDA-LDL and the change in small dense LDL cholesterol in the pemafibrate group. Correlation analysis was performed using Pearson's correlation coefficient. MDA-LDL, malondialdehyde-modified low-density lipoprotein; TG, triglycerides; LDL, low-density lipoprotein.

Supplementary Figure S5. The relationship between lipids and mRNA expression in PBMCs in the study group.

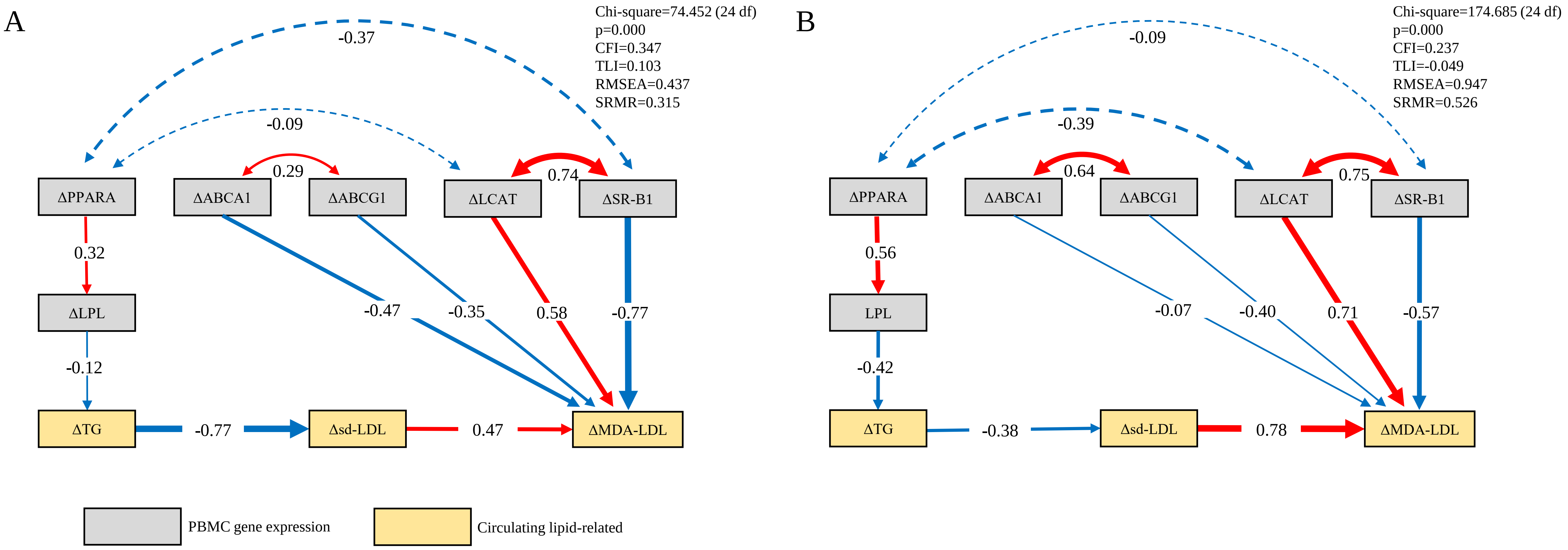

Supplementary Figure S5. The relationship between lipids and mRNA expression in PBMCs in the study group.
Path diagram using structural equation modeling (SEM). A single-direction arrow indicates causality. The numerical value represents the strength of the relationship. The stronger the relationship, the thicker the arrow are displayed.
A: in the Pemafibrate group. B: in the EPA group.
Because the SEM models showed poor fit in both treatment groups, these path diagrams are presented only as exploratory visualizations and should not be interpreted as evidence of confirmed mechanistic pathways.
